# Isothermal and protein-free cascade catalytic hairpin assembly induced-DNAzyme sensing strategy for sensitive miRNA analysis

**DOI:** 10.1038/s41598-025-27979-7

**Published:** 2025-12-23

**Authors:** Yuepeng Zhang, Yuhua Sun, Xueshan Gong, Shuang Zhao, Weiwei Cao, Hongbo Wang, Hao Wang, Xi Zhang, Changwei Du, Zhiguo Chen, Lu Deng

**Affiliations:** Dalian Rehabilitation and Nursing Center of the Joint Logistics Support Force, Liaoning, 116011 China

**Keywords:** Catalytic hairpin assembly, DNAzyme, MiRNA analysis, Fluorescence, Biochemistry, Biological techniques, Biomarkers, Biotechnology, Cancer, Computational biology and bioinformatics, Molecular biology

## Abstract

Sequence-specific microRNAs (miRNAs) represent promising biomarkers for tumor diagnosis, while current detection methods are often limited by stringent temperature requirements and excessive protease dependency. Herein, we present an isothermal, protein-free cascaded amplification circuit for the sensitive analysis of miRNA-21. This strategy, termed CHA-DNAzyme, integrates a miRNA-activated catalytic hairpin assembly (CHA) reaction with a deoxyribozymes (DNAzyme) component. Specifically, the target miRNA-21 initiates the CHA process via a toehold-mediated strand displacement reaction. This reaction unveils a previously sequestered DNAzyme sequence embedded within a hairpin structure, forming an active double-stranded complex. Subsequently, in the presence of Mg²⁺ cofactors, the catalytically activated DNAzyme drives the continuous cleavage of fluorescent reporter hairpins, releasing a measurable fluorescent signal. Implemented in a one-pot format, the CHA-DNAzyme strategy demonstrates excellent sensitivity (with a limit of detection of 8.70 pM), high specificity (the capability to discern single-base mismatches), short sample-to-answer time (approximately 60 min), and robust performance in analyzing serum samples. This approach holds significant potential to facilitate the advancement of miRNA detection tools and support their application in tumor diagnosis and prognosis assessment.

**Supplementary Information:**

The online version contains supplementary material available at 10.1038/s41598-025-27979-7.

## Introduction

MicroRNAs (miRNAs) are a class of endogenous non-coding RNAs, typically 19–25 nucleotides in length^1^. They regulate gene expression primarily by binding to the 3’ untranslated region of target mRNAs, mediating their degradation or translational repression. This regulatory function underpins their critical involvement in tumor progression, invasion, and metastasis^2^. For instance, upregulated miRNA-21 expression is a prominent characteristic of numerous cancer cell types^3^. By targeting key tumor suppressor genes, miRNA-21 promotes tumor cell proliferation, establishing it as a promising biomarker for cancer diagnosis and prognosis assessment^4–6^. However, the inherent characteristics of miRNAs, including high sequence homology among family members, low abundance in biological samples, and susceptibility to degradation by nucleases^7,8^, pose significant challenges for their detection. Current mainstream methodologies, such as Northern blotting, next-generation sequencing, and quantitative real-time polymerase chain reaction, exhibit limitations including labor-intensive protocols, stringent temperature control requirements, and reliance on costly protein enzymes^9–11^. Therefore, a pressing need remains for the development of reliable, highly sensitive, and operationally simple miRNA detection strategies.

Recent years have witnessed significant research efforts directed towards developing isothermal DNA amplification sensing technologies for sensitive miRNA detection. Prominent strategies include loop-mediated isothermal amplification (LAMP), rolling circle amplification (RCA), hybridization chain reaction, and catalytic hairpin assembly (CHA)^12–15^. However, LAMP and RCA methodologies necessitate the involvement of multiple protein enzymes and often entail time-consuming procedures, limiting their suitability for rapid diagnostics in clinical laboratories. In contrast, CHA exemplifies protein-free, isothermal amplification. This strategy leverages target recycling to initiate toehold-mediated strand displacement reactions, driven by favorable free energy changes, resulting in the generation of abundant, stable double-stranded DNA complexes for rapid signal amplification^16^. CHA offers distinct advantages including programmable design, predictable outputs, rapid reaction kinetics, and robust structural stability, facilitating its broad application for detecting diverse biomarkers such as small molecules, proteins, and nucleic acids^17–20^. Furthermore, CHA exhibits excellent modularity, enabling seamless integration with electrochemical, optical, and magnetic transduction platforms for signal readout^21–24^. Nevertheless, the standalone application of CHA for miRNA detection faces challenges. Limitations arise primarily from suboptimal amplification efficiency. Consequently, achieving the sensitivity required for trace miRNA analysis remains difficult. Integrating multiple DNA amplification circuits into cascaded systems represents an increasingly attractive approach to enhance overall detection sensitivity.

Deoxyribozymes (DNAzyme) represent a class of functional nucleic acids capable of catalyzing the sequence-specific cleavage at specific base sites, typically at RNA linkages^25,26^. A DNAzyme comprises a central catalytic core domain flanked by two substrate-binding arms. Through Watson-Crick base pairing, these binding arms hybridize with customizable target sequences, conferring high substrate recognition specificity. Assisted by essential facilitator ions (commonly metal ions), DNAzyme self-assemble into catalytically active structures capable of sustained, multiple-turnover cleavage of their designated substrates^27^. Compared to protein enzymes, DNAzyme offer distinct advantages including significantly lower synthesis costs, high programmability, excellent selectivity, and superior stability. These properties have fostered their widespread application in biosensing^28–31^. Crucially, the compact size of commonly employed DNAzyme (typically 10–30 nucleotides) enables their seamless integration with CHA amplification circuit. This synergistic combination facilitates dual-signal amplification, substantially enhancing both the sensitivity and specificity of miRNA detection assays^32,33^. The prevailing integration approach involves appropriately splitting the DNAzyme catalytic core to form a multicomponent nucleic acid enzyme (MNAzyme), facilitating the incorporation of the separated catalytic components into the hairpin structures of the CHA circuit^34–36^. However, this configuration often leads to reduced cleavage efficiency of the MNAzyme, consequently impairing the overall reaction kinetics^37^. Therefore, developing strategies to incorporate the intact DNAzyme sequence into the CHA amplification circuit represents a crucial direction for enhancing the overall sensing performance.

In this study, we engineered an isothermal, protein-free cascaded amplification strategy, denoted CHA-DNAzyme, by integrating CHA circuit with DNAzyme amplification. This platform achieves accurate and sensitive analysis of miRNA-21 levels. Within this sensing framework, the target miRNA-21 initiates the CHA amplification cascade via a toehold-mediated strand displacement reaction. This process sequentially unfolds hairpins H1 and H2. Crucially, the resultant double-stranded DNA complex facilitates the release of the original miRNA-21 molecule, enabling its participation in subsequent reaction cycles. Concurrently, an intact DNAzyme sequence, previously sequestered within the hairpin structure, becomes accessible. In the presence of Mg²⁺ cofactors, the catalytic activity of the exposed DNAzyme is robustly activated. The activated DNAzyme then drives the continuous cleavage of fluorescently labeled reporter hairpin H3. This enzymatic cleavage event liberates fluorophores, generating a quantifiable fluorescent signal. Through this mechanism, the CHA amplification circuit and the DNAzyme component achieve efficient coupling. Consequently, the inherent dual-signal amplification capability, combining CHA-based target recycling with enzymatic DNAzyme turnover, delivers highly sensitive detection of miRNA-21. The CHA-DNAzyme strategy presents a highly programmable scaffold with demonstrated robustness against interference. This synergy renders it a versatile and operationally streamlined methodology for miRNA detection applications.

## Materials and methods

### Reagents and chemicals

All DNA oligonucleotides and miRNA sequences were custom-synthesized and purified via high-performance liquid Chromatography by Sangon Biotech (Shanghai) Co., Ltd. The detailed sequences are provided in Table 1. The 6× DNA loading buffer, 30% acrylamide/bis-acrylamide gel solution, magnesium chloride hexahydrate (MgCl₂·6 H₂O), and potassium chloride (KCl) were obtained from Solarbio Science & Technology Co., Ltd. (Shanghai, China). Sodium chloride (NaCl) and N, N,N′,N′-Tetramethylethylenediamine (TEMED) were purchased from Aladdin Biochemical Technology Co., Ltd. (China). 5× TBE buffer, ammonium persulfate (APS) substitute, and diethyl pyrocarbonate (DEPC)-treated water were acquired from Beyotime Biotechnology Co., Ltd. (China). Super GelRed™ nucleic acid stain was procured from Biomed Inc. (China). The 20 bp DNA Ladder (Dye Plus) was supplied by Takara Biotechnology Co., Ltd. (Chengdu, China). All chemical reagents were of analytical grade.

**Table 1:** Oligonucleotide sequences used in this study.

Name	Sequence (5′-3′)
miRNA-21	TAGCTTATCAGACTGATGTTGA
H1	TCAACATCAGTCTGATAAGCTACATTCAAATCAGGCTATAGCTTATCAGACT
H2	ATAAGCTATAGCCTGATTTGAATGTAGCTTATCAGACTCATTCAAATCAGGCTAGCTACAACGAACTCAAC
H2-2	ATAAGCTA/iFAMdT/AGCCTGATTTGAATGTAGCTTATCAGACTCATTCAAATCAGGC/iBHQ1dT/AGCTACAACGAACTCAAC
H3	FAM- GTGCGGTGCCTGGGATGGTTGAGT/rA//rU/GATTTGAATTA GTGGGCACCGCAC-BHQ1
miRNA-21-S	TAGCTTATCTGACTGATGTTGA
miRNA-21-T	TAGCTTATCTGAGTGATGTTGA
miRNA-122	TGGAGTGTGACAATGGTGTTTG
miRNA-410	AATATAACACAGATGGCCTGT

### Preparation of DNA hairpin

The CHA-DNAzyme sensing strategy implemented in this work comprises hairpin probes H1, H2, and H3, along with the target molecule, miRNA-21. Prior to experimentation, synthesized oligonucleotide dry powders were reconstituted in DEPC-treated water to a stock concentration of 2 µM. To ensure proper formation of the stem-loop secondary structures, hairpin probes H1, H2, and H3 underwent a thermal annealing protocol. Specifically, the probes were heated to 95 °C for 5 min and then gradually cooled to room temperature at a controlled rate of 1 °C/min. These annealed probes were subsequently employed in all assays. Hairpin H3 incorporates a fluorophore-quencher pair (FAM as the fluorophore and BHQ1 as the quencher). Consequently, solutions containing H3 were protected from light exposure at all stages to preserve the quenching efficiency and minimize background fluorescence.

### Native polyacrylamide gel electrophoresis

The feasibility of the CHA-DNAzyme sensing strategy was validated using native polyacrylamide gel electrophoresis (PAGE). For each sample, 5 µL was combined with 1 µL of 6× DNA loading buffer. These mixtures were then loaded onto a 12% native polyacrylamide gel. Electrophoresis was performed using a gel electrophoresis unit (Bio-Rad Laboratories, USA) submerged in 1× TBE running buffer (89 mM Tris-borate, 2 mM EDTA, pH 8.3). Separation was carried out at a constant voltage of 80 V for 70 min. Post-electrophoresis, the gel was immersed in a diluted solution of Super GelRed™ nucleic acid stain for 20 min. Gel images were subsequently captured and documented using a Gel Doc™ EZ system equipped with Image Lab™ imaging and analysis software (Bio-Rad Laboratories, USA).

### Fluorescence detection

Detection reactions were performed in TNK buffer (5 mM KCl, 140 mM NaCl, 10 mM Tris-HCl, pH 7.6). Target miRNA-21 solutions were introduced into a mixture containing 100 nM hairpin H1 and 100 nM hairpin H2. This solution was incubated for 20 min with gentle agitation to facilitate target recognition and initial complex formation. Following this addition, the reaction mixture was supplemented with 30 mM Mg²⁺ and 600 nM fluorescent reporter hairpin H3. The complete assay mixture was then incubated at room temperature (25 °C) for 60 min to allow the cascade amplification and signal generation processes to proceed. To evaluate assay sensitivity, reactions were conducted using miRNA-21 concentrations spanning a range from 0 pM to 5 nM (specifically: 0, 10, 50, 100, 500 pM, 1 nM, and 5 nM). For specificity assessment, the response to alternative miRNA sequences and base mismatched variants of miRNA-21 was analyzed. Both control reactions proceeded for 60 min at 25 °C. To evaluate the superior performance advantage of the CHA-DNAzyme strategy, an isolated CHA strategy was performed. miRNA-21 was added to a mixture of 100 nM H1 and 100 nM H2-2. The control reactions proceeded for 90 min at 25 °C. Real-time fluorescence monitoring was performed at an emission wavelength of 520 nm using a multimode plate reader (Molecular Devices, USA). Fluorescence spectra were recorded using a Hitachi F-7000 fluorescence spectrophotometer (Hitachi High-Technologies, Japan).

### Detection of miRNA-21 in serum samples

The normal human serum samples were obtained from Sigma-Aldrich. This serum matrix was supplemented with predetermined concentrations of synthetic miRNA-21. The spiked samples were subsequently interrogated using our developed CHA-DNAzyme sensing platform to quantify target miRNA-21 levels within the complex biological matrix. Fluorescence emission spectra were recorded following the reaction using a Hitachi F-7000 spectrofluorometer.

## Results and discussion

### Principle of the CHA-DNAzyme sensing strategy

**Fig. 1:**
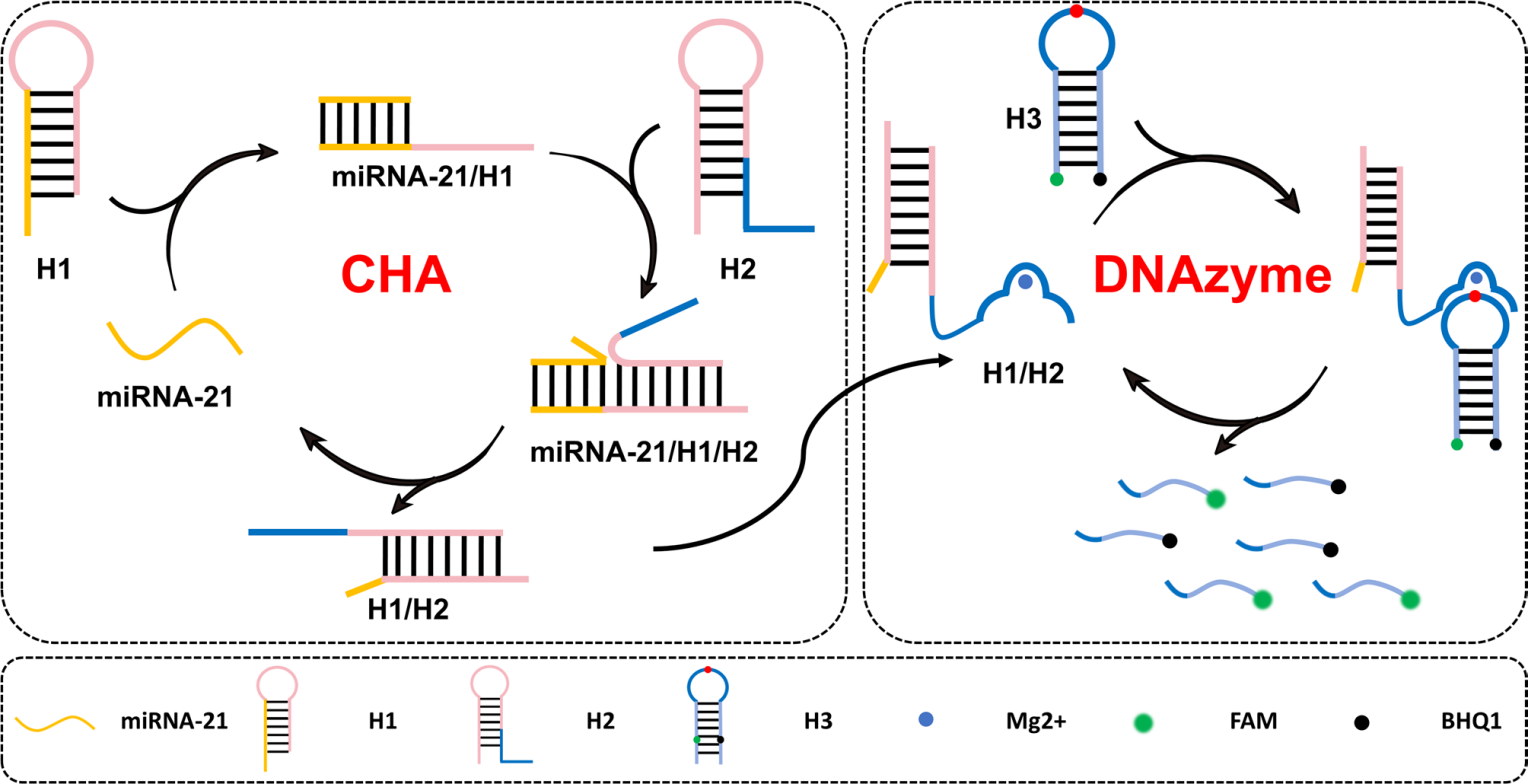
Schematic diagram of CHA-DNAzyme sensing strategy for miRNA-21 analysis.

Figure 1 illustrates the detection principle of the CHA-DNAzyme sensing strategy, which integrates a CHA circuit with DNAzyme amplification. Hairpin H1 serves as the initiator for the CHA circuit and is specifically activated by hybridization with miRNA-21. Hairpin H2 incorporates the DNAzyme sequence partially sequestered within its stem region. Due to this sequestration preventing correct secondary structure folding, the DNAzyme remains catalytically inactive even in the presence of the Mg²⁺ cofactor. Hairpin H3, the reporter probe, is functionalized with a fluorophore (FAM) and a quencher (BHQ1) positioned within its stem. Upon proper folding into the hairpin conformation, efficient fluorescence resonance energy transfer (FRET) between FAM and BHQ1 minimizes background fluorescence. In the presence of miRNA-21, it hybridizes to the toehold domain of H1, initiating a strand displacement reaction that unfolds H1. This forms a miRNA-21/H1 complex and exposes a single-stranded overhang. This exposed overhang subsequently hybridizes with the complementary toehold domain of H2, displacing its stem to form an H1/H2 duplex complex. Crucially, this process releases the miRNA-21 target, enabling its reuse to initiate multiple CHA amplification cycles. Concurrently with H1/H2 duplex formation, the DNAzyme sequence is fully liberated. In the presence of Mg²⁺, the DNAzyme adopts its catalytically active structure. Utilizing its substrate recognition arms, the activated DNAzyme specifically binds to H3 and cleaves the RNA linkage embedded within its loop. This cleavage event disrupts FRET, leading to fluorescence signal recovery. The DNAzyme then dissociates from the cleaved H3 product, allowing it to bind and cleave additional intact H3 molecules. This integrated strategy achieves miRNA-21-mediated dual-amplification (CHA recycling and DNAzyme catalysis) using only three hairpin components, circumventing the need for complex reagents or multi-step procedures. This design significantly enhances detection efficiency while establishing a cost-effective platform.

### Electrophoretic and spectrofluorometric validation of CHA-DNAzyme functionality

**Fig. 2:**
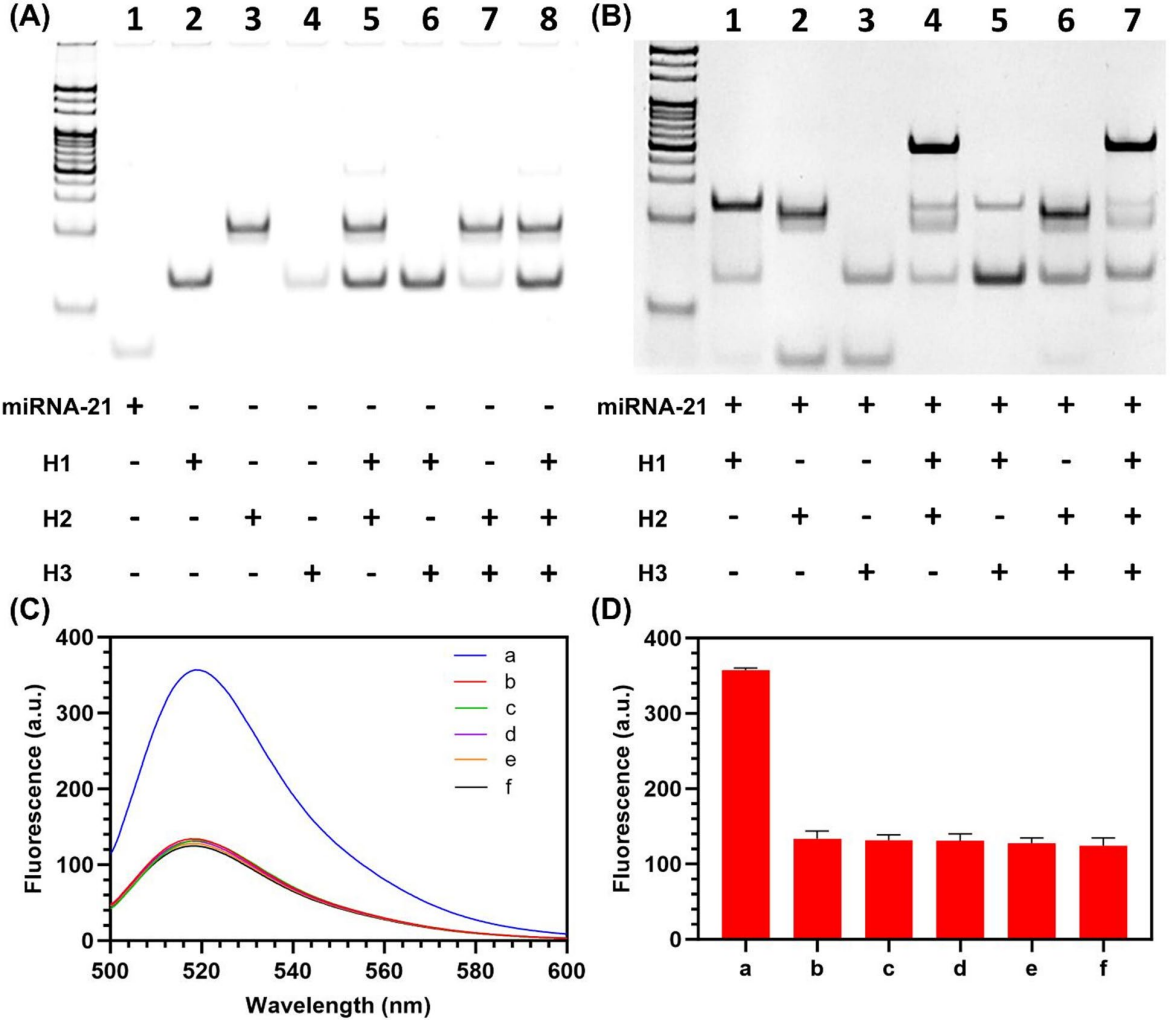
Assembly and feasibility verification of CHA-DNAzyme sensing strategy. (**A**,**B**) PAGE image of the proposed strategy. Original gels are presented in Supplementary Figure S1 and S2. (**C**) The fluorescence spectra of different element combinations in the wavelength range of 500 ~ 600 nm. a: miRNA-21 + H1 + H2 + H3 (with Mg^2+^); b: miRNA-21 + H1 + H2 + H3 (without Mg^2+^); c: miRNA-21 + H2 + H3; d: H1 + H2 + H3; e: H2 + H3; f: H3. The concentration of miRNA-21 was 5 nM. **D** The fluorescence intensity of (**C**) at 520 nm (*N* = 3).

PAGE was employed to verify the synthesis efficiency and functional feasibility of the CHA-DNAzyme sensing components. As depicted in Fig. 2A, individual samples of miRNA-21, H1, H2, and H3 (lanes 1–4) exhibited discrete bands, confirming high purity and absence of synthetic byproducts. The ternary mixture of H1, H2, and H3 incubated without miRNA-21 (lane 8) maintained stable hairpin conformations with negligible nonspecific assemblies, indicating minimal signal leakage inherent to the system. Figure 2B demonstrates that upon introduction of miRNA-21 (lane 4), hybridization with H1 and H2 generated a distinct lower-mobility band, confirming successful CHA circuit assembly. Control experiments revealed that incubating miRNA-21 with H2 and H3 in the absence of H1 (lane 6) preserved the structural integrity of both hairpins, validating effective sequestration of the DNAzyme sequence within H2’s stem and suppression of its catalytic function. Conversely, the complete reaction mixture containing miRNA-21, H1, H2, H3, and Mg²⁺ (lane 7) produced a band pattern analogous to lane 4, accompanied by significantly reduced intensity of the H3 band and the emergence of lower molecular weight species, directly evidencing DNAzyme activation and sustained catalytic cleavage of H3.

Fluorescence analysis further corroborated the sensing strategy’s validity. Figure 2C and D illustrate that the mixture containing only H1, H2, and H3 generated background fluorescence intensity comparable to isolated H3. Crucially, introducing miRNA-21 without Mg²⁺ failed to elicit significant fluorescence enhancement, confirming the structural stability of the hairpin probes and consequent low background signal—a prerequisite for achieving high detection sensitivity. The essential role of Mg²⁺ as a DNAzyme cofactor was unequivocally demonstrated, as its inclusion triggered immediate and substantial fluorescence amplification. Collectively, these electrophoretic and fluorometric results confirm the operational feasibility of the integrated CHA-DNAzyme platform for miRNA-21 detection.

### Demonstrating superior amplification via CHA-DNAzyme cascades

**Fig. 3:**
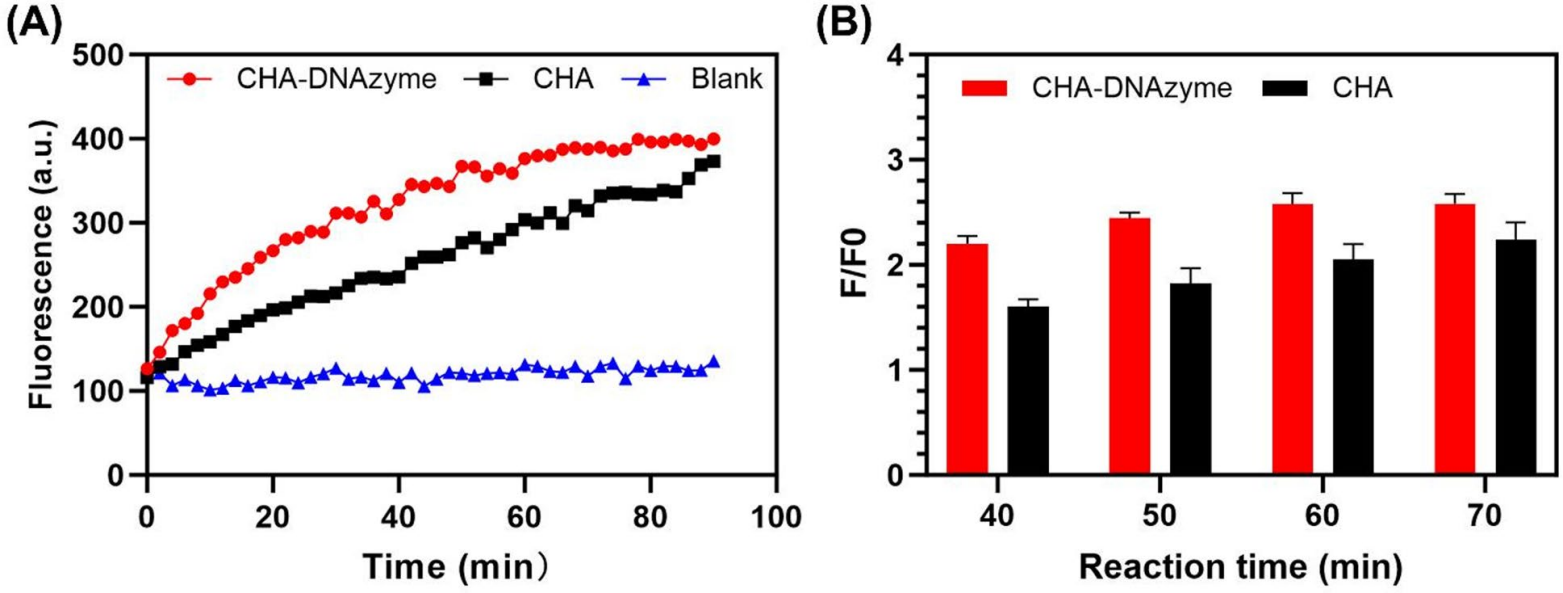
(**A**) Comparison of reaction kinetics of the proposed CHA-DNAzyme strategy and CHA strategy. (**B**) The F/F0 ratio of the proposed CHA-DNAzyme strategy and CHA strategy at different reaction time (*N* = 3). The concentration of miRNA-21 was 5 nM. F represents the fluorescence intensity at different reaction time points and F0 represents the initial fluorescence intensity.

To establish the enhanced amplification capability conferred by the dual-signal mechanism of the CHA-DNAzyme strategy, we directly compared its reaction kinetics with those of an isolated CHA cascade when activated by identical miRNA-21 concentrations. As evidenced in Fig. 3A, both systems exhibited progressive fluorescence enhancement over time. However, the CHA-DNAzyme platform demonstrated significantly accelerated signal growth kinetics, plateauing at approximately 60 min. Kinetic analysis further revealed distinct behaviors in the normalized fluorescence intensity ratio (F/F0). While the F/F0 ratio for the CHA-DNAzyme system stabilized beyond 60 min, the CHA cascade exhibited a persistent increase throughout the monitored period (Fig. 3B). Consequently, endpoint fluorescence measurements at 60 min were selected for subsequent miRNA-21 quantification. These kinetic profiles substantiate that the integrated CHA-DNAzyme platform, leveraging cascade signal amplification, achieves rapid and efficient target signal multiplication, thereby contributing to its enhanced detection sensitivity.

### Optimization of the critical experimental conditions

To establish optimal sensing performance of the CHA-DNAzyme strategy, key reaction conditions were systematically evaluated. Given the significant temperature dependence of both the free-energy-driven CHA amplification and DNAzyme cleavage activity, reaction temperature.

**Fig. 4:**
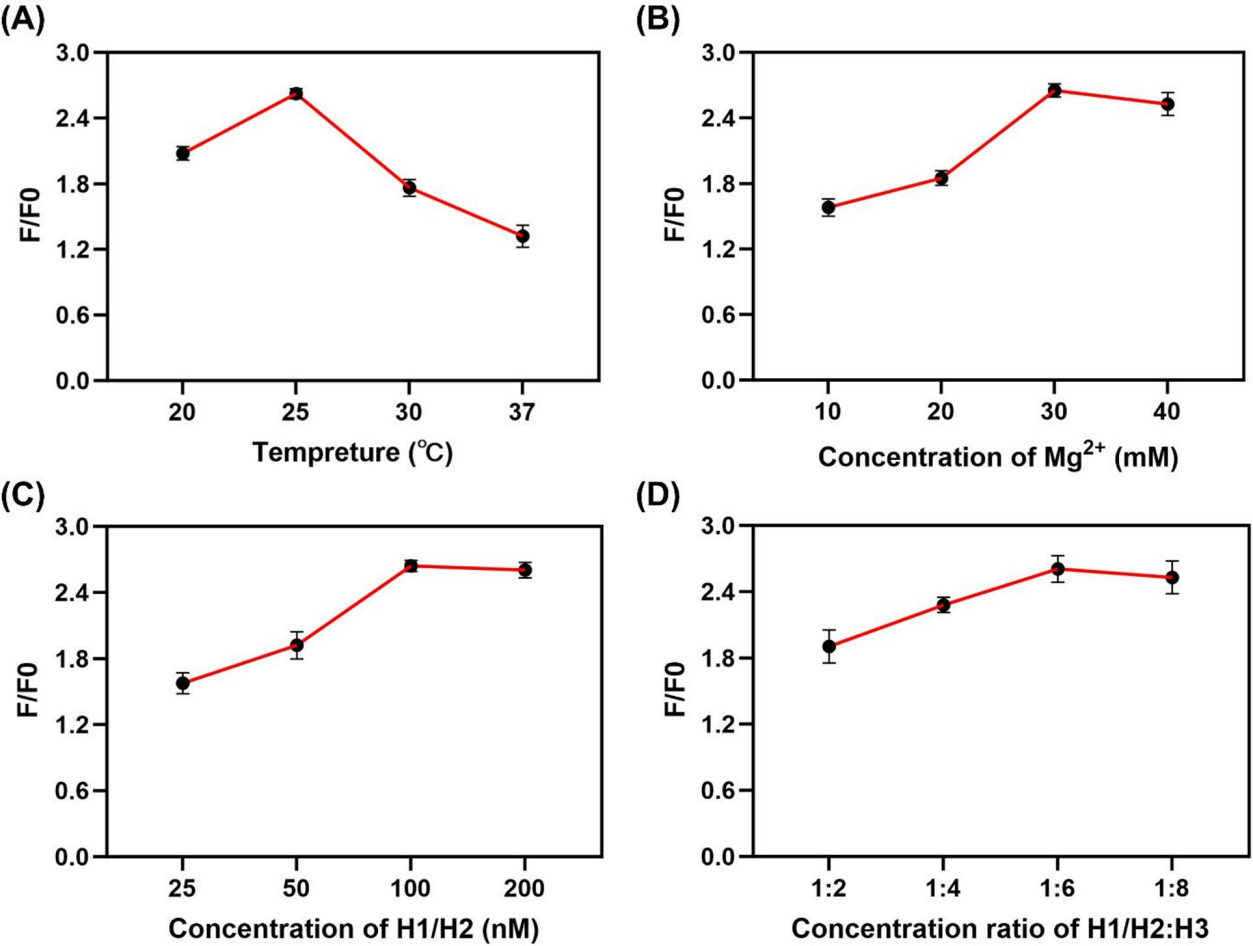
Optimization of the proposed CHA-DNAzyme sensing strategy. The effect of the reaction temperature (**A**), the concentration of Mg^2+^ (**B**), the concentration of H1/H2 (H1:H2 = 1:1) (**C**) and the concentration ratio of H1/H2 to H3 (**D**). The concentration of miRNA-21 was 5 nM (*N* = 3).

was prioritized. As shown in Fig. 4A, the normalized fluorescence ratio (F/F₀) reached its maximum at 25 °C, decreasing substantially with elevated temperatures. This observation may arise from increased destabilization of metastable hairpin conformations at higher temperatures, potentially enhancing nonspecific assemblies and consequent background signal leakage^38,39^. Subsequently, the concentration of Mg²⁺, an essential DNAzyme cofactor critically modulating its catalytic activation, was optimized. Incremental elevation of Mg²⁺ concentration progressively enhanced the F/F₀ ratio, achieving a maximum at 30 mM (Fig. 4B). This concentration (30 mM Mg²⁺) was consequently employed for all subsequent assays. The influence of initiator (H1) and amplifier (H2) hairpin concentrations was then investigated. Figure 4C demonstrated a concentration-dependent increase in F/F₀ ratio for H1 and H2, plateauing at 100 nM. This behavior likely reflects the inherent dependence of miRNA-21-triggered CHA amplification on the stochastic collision frequency between the target, H1, and H2 within the reaction milieu. Higher probe concentrations increase this collision probability, thereby improving reaction kinetics. Finally, the concentration of the reporter hairpin H3, serving dual roles as the fluorogenic substrate and DNAzyme cleavage target, directly governs the detectable signal amplitude. Increasing H3 concentration rapidly amplified the F/F₀ ratio (Fig. 4D), with signal saturation observed at a 1:6 molar ratio of H1/H2 to H3. This optimal stoichiometry was thus adopted for further experiments.

### The performance of the proposed CHA-DNAzyme strategy

**Fig. 5:**
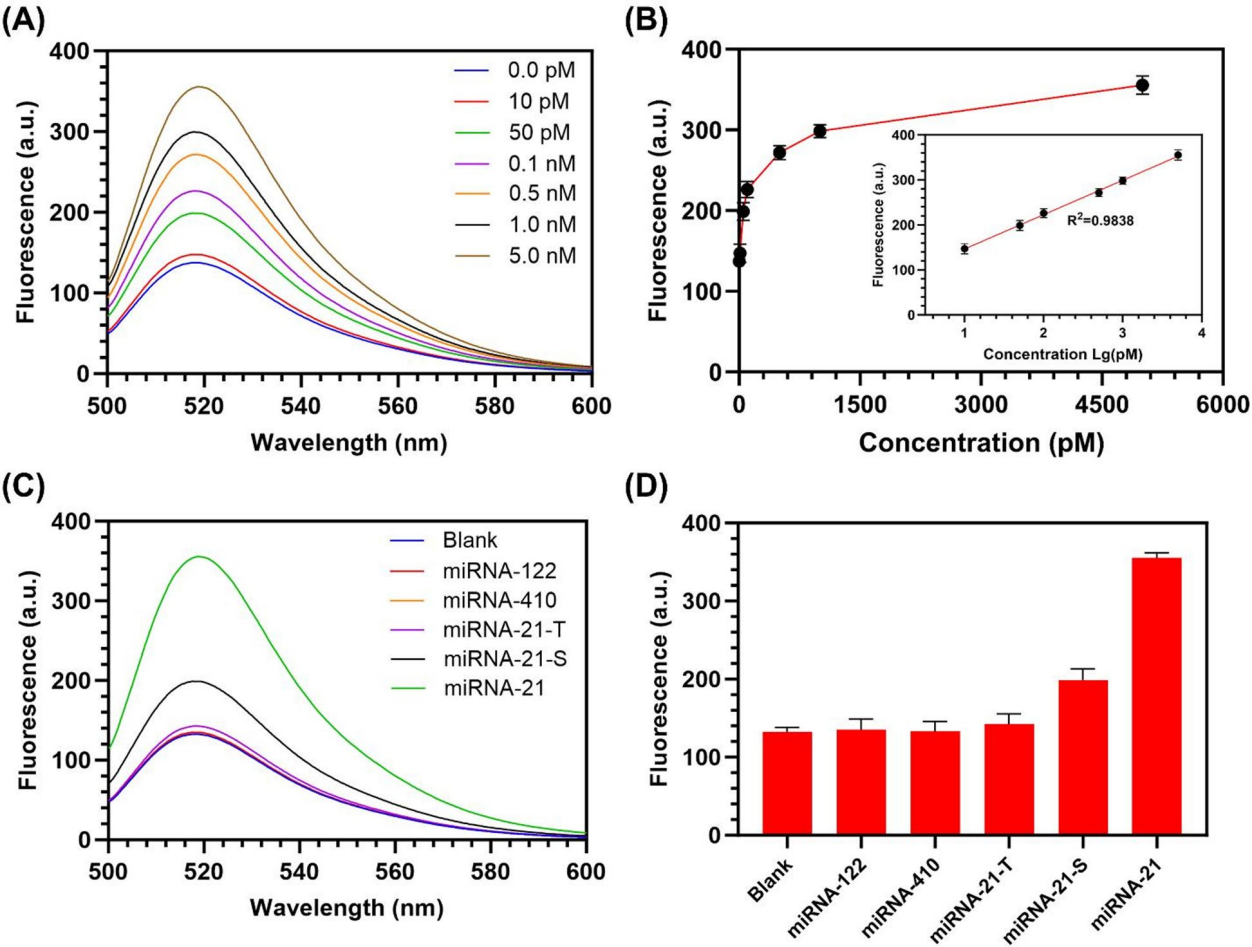
Evaluation of the sensing performance of the proposed strategy. (**A**) The fluorescence spectra of the CHA-DNAzyme sensing strategy in the presence of different concentrations of miRNA-21. (**B**) Calibration curve of the fluorescence intensity versus the logarithm of the miRNA-21 concentration (10 pM to 5 nM). (**C**) The fluorescence spectra of the CHA-DNAzyme sensing strategy initiated by miRNA-21, single-base mutated miRNA-21 (miRNA-21-S), two-base mutated miRNA-21 (miRNA-21-T), miRNA-410 and miRNA-122. The concentration miRNA was 5 nM. (**D**) The fluorescence intensity of (**C**) at 520 nm (*N* = 3).

Under the optimized conditions detailed previously, the sensitivity of the CHA-DNAzyme sensing strategy was evaluated by introducing synthetic miRNA-21 across a concentration gradient (10 pM to 5 nM) and monitoring the resultant fluorescence signal. As depicted in Fig. 5A, fluorescence intensity at 520 nm exhibited a concentration-dependent increase. Quantitative analysis revealed a robust linear correlation (R² = 0.9838) between the logarithmic miRNA-21 concentration and the fluorescence intensity over the entire tested range (10 pM to 5 nM) (Fig. 5B). The limit of detection was calculated to be approximately 8.70 pM, based on the 3σ criterion (three times the standard deviation of blank measurements). This significantly enhanced sensitivity, surpassing that achieved by standalone CHA or DNAzyme strategies, is attributed to the synergistic coupling of minimal background leakage and the dual amplification cascade.

Furthermore, assay specificity was rigorously assessed against potential interferents, including single-nucleotide variant miRNA-21 (miRNA-21-S), double-nucleotide variant miRNA-21 (miRNA-21-T), miRNA-122, and miRNA-410. Under identical assay conditions, these non-specific targets generated fluorescence intensities markedly lower than the true miRNA-21 signal, approaching levels equivalent to the background (miRNA-free) control. While miRNA-21-S induced a minor fluorescence increase, the signal enhancement remained less than one-third of that elicited by an equivalent concentration of perfectly matched miRNA-21 (Figs. 5C, D). These results demonstrate that the CHA-DNAzyme cascade initiates exclusively in the presence of its highly specific target substrate, confirming exceptional specificity and strong resistance to miRNA interferents.

### Detection of miRNA-21 in serum samples

**Table 2:** Detection of miRNA-21 in human serum samples (*N* = 3).

Samples	Added (pM)	Found (pM)	Recovery (%)	RSD (%)
1	50	52.37	104.74	5.38
2	100	104.98	104.98	5.84
3	1000	962.83	96.28	4.37

Building upon prior experimental validation, we assessed the efficacy of the CHA-DNAzyme sensing platform for detecting miRNA-21 within a clinically relevant matrix. Synthetic miRNA-21 was spiked into human serum samples at three distinct concentrations (50 pM, 100 pM, and 1000 pM). As summarized in Table 2, analysis of triplicate measurements for each spiked sample yielded relative standard deviation (RSD) values ranging from 4.37% to 5.84% and recovery rates between 96.28% and 104.98%. The close agreement between the spiked (actual) and measured concentrations of miRNA-21 confirms the accuracy and reliability of the assay within this complex biological fluid. This robust performance in serum substantiates the platform’s potential for clinical application in quantifying circulating miRNA-21, positioning it as a promising tool to support precise cancer diagnostics and prognostic assessment.

## Conclusion

In summary, we have developed an isothermal, protein-free miRNA detection platform leveraging synergistic integration of CHA circuit with DNAzyme amplification for the accurate and sensitive quantification of miRNA-21. This strategy efficiently converts the inherently labile miRNA signal into a stable double-stranded DNA complex via the CHA cascade, significantly mitigating signal loss due to miRNA degradation. The assay’s exceptional analytical sensitivity and specificity, enhanced by the dual-amplification mechanism, enable reliable detection of low-abundance miRNA targets within complex serum matrices. Critically, the entire reaction is performed isothermally in a one-pot format using only three DNA hairpin components, eliminating the requirement for expensive protein enzymes. This design inherently streamlines operational workflows while reducing both instrumentation dependency and assay costs. Consequently, this CHA-DNAzyme platform holds significant promise for clinical translation, paving the way for improved precision diagnostics in oncology based on miRNA profiling.

## Supplementary Information

Below is the link to the electronic supplementary material.

Supplementary Material 1

## Data Availability

The datasets used and/or analyzed during the current study available from the corresponding author on reasonable request.
