# Supplementary material for: Isothermal and protein-free cascade catalytic hairpin assembly induced-DNAzyme sensing strategy for sensitive miRNA analysis
Source: Sci Rep. 2025 Dec 23;15:44359. doi: 10.1038/s41598-025-27979-7 (PMC12728169; doi:10.1038/s41598-025-27979-7)
Supplement: Supplementary file 1 — Supplementary Material 1 [file 41598_2025_27979_MOESM1_ESM.docx]

**Supplementary Information**

**Isothermal and protein-free cascade catalytic hairpin assembly induced-DNAzyme sensing strategy for sensitive miRNA analysis**

Yuepeng Zhang^1^, Yuhua Sun^1^, Xueshan Gong, Shuang Zhao, Weiwei Cao, Hongbo Wang, Hao Wang, Xi Zhang, Changwei Du^*^, Zhiguo Chen^*^, Lu Deng^*^

*Dalian Rehabilitation and Nursing Center of the Joint Logistics Support Force, Liaoning, 116011, China*

***Corresponding authors:** Lu Deng, E-mail: zhangjm3050@163.com; Zhiguo Chen, E-mail: 178249485@qq.com; Changwei Du, E-mail: 316649338@qq.com.

^1^ These three authors contributed equally to this work.

**Supplementary Figures**


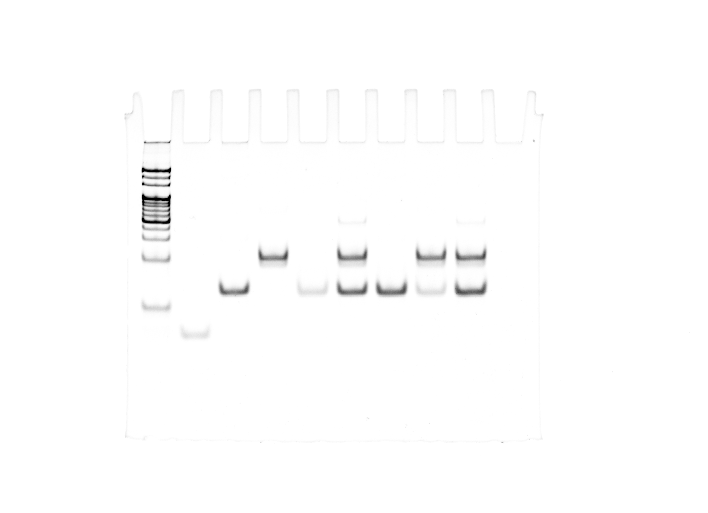


**Supplementary S1.** The original gel of Figure 2A.


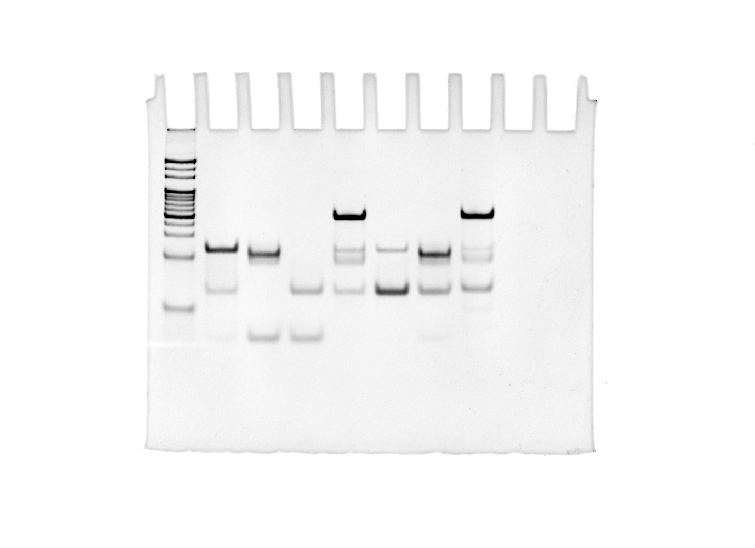


**Supplementary S2.** The original gel of Figure 2B.
